# Triglyceride-glucose index is associated with quantitative flow ratio in patients with acute ST-elevation myocardial infarction after percutaneous coronary intervention

**DOI:** 10.3389/fcvm.2022.1002030

**Published:** 2022-09-08

**Authors:** Bingyan Yu, Yuhao Mo, Xiangming Hu, Weimian Wang, Jieliang Liu, Junguo Jin, Ziheng Lun, Ci Ren Luo Bu, Haojian Dong, Yingling Zhou

**Affiliations:** ^1^School of Medicine, South China University of Technology, Guangzhou, China; ^2^Guangdong Provincial Key Laboratory of Coronary Heart Disease Prevention, Department of Cardiology, Guangdong Cardiovascular Institute, Guangdong Provincial People’s Hospital, Guangdong Academy of Medical Sciences, Guangzhou, China; ^3^Nyingchi People’s Hospital, Nyingchi, China

**Keywords:** triglyceride-glucose index, quantitative flow ratio, ST-elevation myocardial infarction, percutaneous coronary intervention, fasting blood glucose

## Abstract

**Background:**

The triglyceride-glucose (TyG) index is a novel marker representing the degree of insulin resistance (IR) and is closely related to cardiovascular diseases. However, the association between the TyG index and vascular function in patients with acute ST-elevation myocardial infarction (STEMI) after percutaneous coronary intervention (PCI) remains unknown.

**Materials and methods:**

This study was a *post hoc* analysis of a multicenter, prospective cohort study. In this study, patients with STEMI who underwent PCI were included, and coronary angiography data were analyzed by Quantitative coronary angiography (QCA) and quantitative flow ratio (QFR). In addition, the TyG index was calculated as follows: Ln [fasting triglyceride (mg/dl) × fasting blood glucose (mg/dl) × 1/2]. According to the post-PCI QFR, patients were divided into two groups: post-PCI QFR ≤ 0.92 group and post-PCI QFR > 0.92 group. Construction of logistic regression model to explore the relationship between the TyG index and post-PCI QFR.

**Results:**

A total of 241 STEMI patients were included in this study. Compared with patients in the post-PCI QFR > 0.92 group, the TyG index was higher in the post-PCI QFR ≤ 0.92 group. Logistic regression model showed that after adjusting for other confounding factors, the TyG index was positively correlated with the risk of post-PCI QFR ≤ 0.92 (OR = 1.697, 95% CI 1.171–2.460, *P* = 0.005). Restricted cubic splines showed the cutoff value of TyG index associated with post-PCI QFR ≤ 0.92 risk was 9.75.

**Conclusion:**

The TyG index was associated with the risk of post-PCI QFR ≤ 0.92 in STEMI patients. The risk of post-PCI QFR ≤ 0.92 increased when the TyG index exceeded 9.75.

## Introduction

Acute ST-elevation myocardial infarction (STEMI) remains one of the causes of high mortality. With the development of percutaneous coronary intervention (PCI) technology and secondary prevention treatment strategies, the prognosis of STEMI patients has improved, but some patients still have adverse events after PCI, such as in-stent restenosis and unintended revascularization, partly explained by residual ischemia of the coronary arteries (1–3). Physiological assessment after PCI, such as quantitative flow ratio (QFR) analysis, can be used as an effective means to quantify residual coronary ischemia (4–6). Studies have confirmed that poor post-PCI QFR is associated with a poorer prognosis, but the susceptibility factors affecting poor postoperative QFR remain unclear (7–9). Therefore, identifying risk factors for poor postoperative QFR has important clinical significance for reducing the risk of coronary residual ischemia.

Insulin resistance (IR) is a recognized indicator of systemic inflammation and metabolic disorders, is closely related to atherosclerotic cardiovascular disease, and is a high-risk factor for diabetes mellitus (DM) and cardiovascular disease (10, 11). The current methods for assessing IR include the hyperinsulinemia-euglycemic clamp and homeostasis model assessment-estimated IR (HOMA-IR), but their clinical use is limited due to time-consuming and expensive (12, 13). The triglyceride-glucose (TyG) index based on fasting blood glucose (FBG) and triglyceride (TG) has become an effective surrogate index for evaluating IR because of its rapidity and simplicity (14–16). More and more studies have found that the TyG index is not only significantly associated with the risk of atherosclerosis, DM, and coronary artery disease (CAD), but its elevated levels increase the poor prognosis of cardiovascular disease such as in-stent restenosis, atrial fibrillation (17–20).

However, to date, the association between the TyG index and residual coronary ischemia after PCI in STEMI patients has not been explored. Therefore, the aim of this study was to investigate the relationship between the TyG index and post-PCI QFR in STEMI patients.

## Materials and methods

### Study population

This study is a *post hoc* analysis of a multicenter, prospective cohort study (the outcomes in patients with STEMI with high thrombus burden treated by deferred versus immediate stent implantation in primary percutaneous coronary intervention: a prospective cohort study, which was registered at www.chictr.org.cn, ChiCTR1800019923), which was conducted in three cardiovascular centers (Guangdong Provincial People’s Hospital, Guangzhou City; Guangdong Provincial People’s Hospital Zhuhai Hospital, Zhuhai City; and Jiexi County People’s Hospital, Jiexi City) from January 2018 to April 2021. STEMI patients who successfully underwent PCI were included in this study. The main exclusion criteria were: (1) age < 18 years; (2) culprit vessel treated with underwent balloon angioplasty without stents implantation; (3) no fasting blood glucose measurement; (4) history of coronary artery bypass grafting. This study complies with the Declaration of Helsinki and was approved by the Ethics Committee of Guangdong Provincial People’s Hospital. Written consent was obtained from all participants for this study.

### Data collection and definition

Data regarding patient demographics, clinical characteristics, echocardiography, coronary angiography and laboratory results were obtained from electronic medical records. Blood samples for analysis were drawn after an overnight fast (> 10 h). High-density lipoprotein cholesterol (HDL-C), low-density lipoprotein cholesterol (LDL-C), total cholesterol (TC), and TG levels were detected using AU5800 spectrophotometer (Beckman Coulter, United States) *via* colorimetry or immunoturbidimetry. Triglyceride-glycemic index was calculated by the formula Ln [fasting TG (mg/dL) × FBG (mg/dL)/2].

Hypertension was defined as systolic blood pressure ≥ 140 mmHg and/or diastolic blood pressure ≥ 90 mmHg (21). The diagnostic criteria for DM were FBG ≥ 7.0 mmol/L or random blood glucose > 11.1 mmol/L, and glycosylated hemoglobin ≥ 6.5% (22).

### Quantitative coronary angiography and quantitative flow ratio analysis

Quantitative coronary angiography (QCA) and QFR analyses were performed before and after PCI in culprit vessel. QCA and QFR before PCI referred to QCA and QFR after pretreatment with balloon. The measurement of QCA needs to select two parts with an end-diastolic projection angle > 25° and no shortening or overlapping part for coronary 3D reconstruction. The measurement contents include reference vessel diameter, lesion length, minimum lumen diameter, and diameter stenosis rate. QFR assessment was performed on all criminal vessels that underwent PCI. The measurement of QFR is based on three-dimensional QCA analysis and frame technique analysis without drug congestion. QFR calculations were performed by two experienced technicians (Independent Core Laboratory, Shanghai, China) using prototype software (AngioPlus Core, Pulse Medical Imaging Technology, Shanghai, China).

### Statistical analysis

Continuous variables were described as mean ± standard deviation, and categorical variables were described as counts and percentages. For continuous variables, use the *T*-test or the Mann–Whitney U test, and for categorical variables, use the Chi-square test or Fisher’s exact test to compare the differences between the two groups. Logistic regression analysis explored the relationship between the TyG index and post-PCI QFR by odds ratio (OR) with a 95% confidence interval (CI). Factors that might affect post-PCI QFR in baseline data were included in the regression equation to control for the influence of confounding factors. Model 1 adjusted for age, sex, body mass index (BMI), and model 2 adjusted for left ventricular ejection fraction (LVEF), smoking, hypertension, DM, previous myocardial infarction, and creatinine based on model 1. Model 3 continued to adjust the culprit vessel, stent length, in-stent minimum lumen diameter, and in-stent diameter stenosis rate based on model 2. Restricted cubic splines were used to explore the association between the TyG index and the risk of post-PCI QFR ≤ 0.92 on a continuous scale. Sensitivity analysis was performed to evaluate the association between the TyG index and the risk of post-PCI QFR ≤ 0.92 in the non-chronic total occlusion (CTO) subgroup, and the results are presented in the Supplementary material. All statistical analyses were performed on IBM SPSS Statistics 26 and R language 4.1.2. Two-sided *P* < 0.05 was considered statistically significant.

## Results

### Baseline characteristics

A total of 241 STEMI patients were included in this study. As shown in Table 1, among the included patients, 151 patients had optimal PCI results (post-PCI QFR > 0.92), while 90 patients had suboptimal PCI results (post-PCI QFR ≤ 0.92). Of the total population, 84.2% were male, and the mean age was 62 years. The prevalence of smoking, hypertension, DM, and family history of CAD, previous myocardial infarction, and peripheral vascular disease were 45.6, 44.0, 13.7, 1.7, 11.2, and 2.1%, respectively. The distribution of STEMI types differed between the two groups, with patients in the post-PCI QFR ≤ 0.92 group having a higher proportion of anterior STEMI, whereas patients in the post-PCI QFR > 0.92 group had a higher proportion of non-anterior STEMI. Compared with the Post-PCI QFR > 0.92 group, the patients in the post-PCI QFR ≤ 0.92 group showed a higher FBG and TyG index, but a lower LVEF. Age, male ratio, BMI, smoking, hypertension, DM, family history of CAD, previous myocardial infarction, incidence of peripheral vascular disease, time from onset to door, TG, TC, HDL-C, LDL-C, peak creatine kinase isoenzyme MB (CK-MB), peak troponin T (TnT), and Peak NTpro-brain natriuretic peptide (BNP) were not different between the two groups. The QCA and QFR analysis for all patients was listed in Table 2. Post-PCI QFR ≤ 0.92 and post-PCI QFR > 0.92 groups had mean post-PCI QFR of 0.87 and 0.96, respectively. Patients in the suboptimal PCI result group had a higher proportion of the culprit vessel in the left anterior descending artery, a smaller in-stent minimum lumen diameter, and a greater in-stent diameter stenosis rate.

**TABLE 1:** Baseline characteristics.

	Total (*N* = 241)	Post-PCI QFR ≤ 0.92 (*n* = 90)	Post-PCI QFR > 0.92 (*n* = 151)	*P*-value
Age, years	62 ± 12	61 ± 11	63 ± 12	0.184
Male	203 (84.2)	78 (86.7)	125 (82.8)	0.469
BMI, kg/m^2^	24.18 ± 3.53	24.28 ± 3.78	24.11 ± 3.38	0.721
LVEF, %	49.3 ± 11.4	46.7 ± 12.0	50.9 ± 10.8	0.006*
**Medical history, *n* (%)**				
Smoking	110 (45.6)	38 (42.2)	72 (47.7)	0.426
Hypertension	106 (44.0)	42 (46.7)	64 (42.2)	0.592
DM	33 (13.7)	13 (14.4)	20 (13.2)	0.847
Family history of CAD	4 (1.7)	1 (1.1)	3 (2.0)	1.000
Previous myocardial infarction	27 (11.2)	10 (11.1)	17 (11.3)	1.000
Peripheral vascular disease	5 (2.1)	0 (0)	5 (3.3)	0.160
O to D, hours	9.23 ± 11.58	9.25 ± 13.11	9.23 ± 10.61	0.990
STEMI types				0.002*
Anterior	124 (51.5)	58 (64.4)	66 (43.7)	
Non-anterior	117 (48.5)	32 (35.6)	85 (56.3)	
**Laboratory tests**				
FBG, mmol/L	8.78 ± 3.98	9.62 ± 4.49	8.27 ± 3.57	0.016*
Creatine, mmol/L	86.91 ± 72.92	80.91 ± 34.01	90.49 ± 88.25	0.325
TG, mmol/L	2.20 ± 1.75	2.42 ± 1.94	2.07 ± 1.62	0.139
TC, mmol/L	4.58 ± 1.74	4.56 ± 1.91	4.59 ± 1.63	0.889
HDL-C, mmol/L	1.07 ± 0.28	1.04 ± 0.22	1.09 ± 0.30	0.182
LDL-C, mmol/L	3.37 ± 0.93	3.46 ± 1.07	3.31 ± 0.84	0.250
Peak CK-MB, U/L	284.56 ± 388.97	249.60 ± 216.61	305.39 ± 461.59	0.282
Peak TnT, pg/mL	8919.4 ± 45512.1	5258.7 ± 3403.3	10129.3 ± 57432.2	0.303
Peak NT-proBNP, pg/mL	3201.0 ± 8043.9	2916.1 ± 4866.0	3370.8 ± 9455.2	0.672
TyG index	9.31 ± 0.79	9.50 ± 0.82	9.21 ± 0.75	0.005*

PCI, percutaneous coronary intervention; QFR, quantitative flow ratio; BMI, body mass index; LVEF, left ventricular ejection fraction; DM, diabetes mellitus; CAD, coronary artery disease; O to D, time from onset to door; STEMI, ST-elevation myocardial infarction; FBG, fasting blood glucose; TG, triglyceride; TC, total cholesterol; HDL-C, high-density lipoprotein cholesterol; LDL-C, low-density lipoprotein cholesterol; CK-MB, peak creatine kinase isoenzyme MB; TnT, troponin T; NT-proBNP, N terminal pro B type natriuretic peptide; TyG, triglyceride-glucose.

Bold term and * indicates statistically significant.

**TABLE 2:** Coronary characteristics.

	Total (*N* = 241)	Post-PCI QFR ≤ 0.92 (*n* = 90)	Post-PCI QFR > 0.92 (*n* = 151)	*P*-value
Culprit vessel				0.008*
LAD	124 (51.5)	58 (64.4)	66 (43.7)	
LCX	14 (5.8)	4 (4.4)	10 (6.6)	
RCA	103 (42.7)	28 (31.1)	75 (49.7)	
**Pre-PCI QCA**				
Reference vessel diameter, mm	2.94 ± 0.86	2.89 ± 0.84	2.97 ± 0.88	0.463
Minimal lumen diameter, mm	1.07 ± 0.59	1.05 ± 0.57	1.08 ± 0.61	0.788
Diameter stenosis, %	63.1 ± 17.1	63.2 ± 16.0	63.0 ± 17.7	0.939
lesion length, mm	14.3 ± 6.5	13.4 ± 5.4	14.8 ± 7.0	0.103
**Post-PCI QCA**				
In-stent reference vessel diameter, mm	3.12 ± 0.61	3.04 ± 0.51	3.17 ± 0.67	0.125
In-stent minimal lumen diameter, mm	2.54 ± 0.59	2.40 ± 0.55	2.63 ± 0.60	0.002*
In-stent diameter stenosis, %	18.6 ± 11.3	21.2 ± 13.5	17.1 ± 9.4	0.012*
Stent length, mm	24.4 ± 16.1	28.0 ± 16.1	30.3 ± 16.1	0.277
Pre-PCI QFR	0.40 ± 0.40	0.37 ± 0.37	0.41 ± 0.41	0.418
Post-PCI QFR	0.93 ± 0.08	0.87 ± 0.11	0.96 ± 0.02	< 0.001*

LAD, left anterior descending artery; LCX, Left circumflex artery; RCA, right coronary artery; QCA, quantitative coronary angiography; PCI, percutaneous coronary intervention; QFR, quantitative flow ratio.

Bold term and * indicates statistically significant.

### Association between the triglyceride-glucose index and post-percutaneous coronary intervention quantitative flow ratio

**FIGURE 1:**
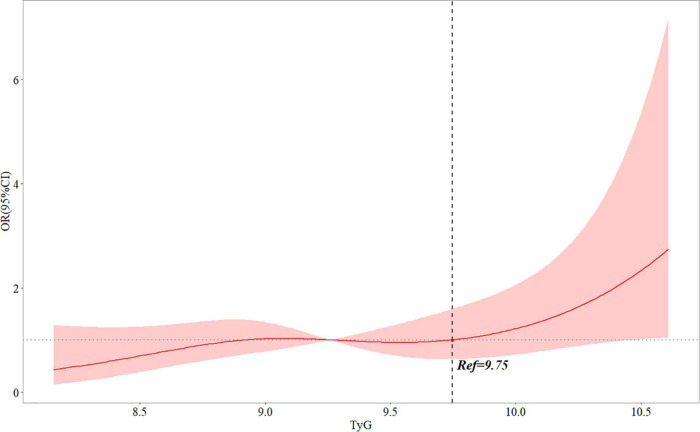
Multivariable adjusted OR for the risk of post-PCI QFR ≤ 0.92 according to levels of TyG index on a continuous scale. Odds ratios and 95% CIs derived from restricted cubic spline regression, with knots placed at the 5th, 35th, 65th, and 95th percentiles of the distribution of TyG index. The reference point for TyG index is located at OR = 1. Ref represents the level of TyG index at increased risk of post-PCI QFR ≤ 0.92. Analyses were adjusted for age, sex, BMI, LVEF, smoking, hypertension, DM, previous myocardial infarction, creatine, culprit vessel, length of stents, in-stent minimal lumen diameter and in-stent diameter stenosis. OR, odds ratio; QFR, quantitative flow ratio; TyG, triglyceride-glucose; BMI, body mass index; LVEF, left ventricular ejection fraction; DM, diabetes mellitus; PCI, percutaneous coronary intervention; CI: confidence interval.

In an unadjusted logistic regression model, the TyG index was positively associated with the risk of post-PCI QFR ≤ 0.92. After adjusting for other factors, the risk of TyG index and post-PCI QFR ≤ 0.92 in model 1 (OR = 1.611, 95% CI 1.142–2.273, *P* = 0.007), model 2 (OR = 1.672, 95% CI 1.177- 2.374, *P* = 0,004), and model 3 (OR = 1.697, 95% CI 1.171–2.460, *P* = 0.005) were still independently associated (Table 3). Furthermore, the relationship between the TyG index and the risk of post-PCI QFR ≤ 0.92 remained significantly associated in the non-CTO population (OR = 1.687, 95% CI 1.162–2.448, *P* = 0.006) (Supplementary Table 3). The relationship between the TyG index and the risk of post-PCI QFR ≤ 0.92 was non-linear in the continuous range of the TyG index (Figure 1). The cutoff value of the TyG index associated with risk of post-PCI QFR ≤ 0.92 was 9.75 (Figure 1).

**TABLE 3:** Association of TyG index with the risk of post-PCI QFR ≤ 0.92 in logistic regression models.

	OR	95% CI	*P*-value
**Unadjusted model**			
TyG, per 1-unit increase	1.611	1.142–2.273	0.007*
**Model 1**			
TyG, per 1-unit increase	1.611	1.142–2.273	0.007*
**Model 2**			
TyG, per 1-unit increase	1.672	1.177–2.374	0.004*
**Model 3**			
TyG, per 1-unit increase	1.697	1.171–2.460	0.005*

Model 1: adjusted for age, sex, and BMI.

Model 2: adjusted for age, sex, BMI, LVEF, smoking, hypertension, DM, previous myocardial infarction, and creatine.

Model 3: adjusted for age, sex, BMI, LVEF, smoking, hypertension, DM, previous myocardial infarction, creatine, culprit vessel, length of stents, in-stent minimal lumen diameter and in-stent diameter stenosis.

TyG, triglyceride-glucose; OR, odds ratio; CI, confidence interval; BMI, body mass index; LVEF, left ventricular ejection fraction; DM, diabetes mellitus; PCI, percutaneous coronary intervention; QFR, quantitative flow ratio.

Bold term and * indicates statistically significant.

### Association between the triglyceride-glucose index and post-percutaneous coronary intervention quantitative flow ratio in different subgroups

**FIGURE 2:**
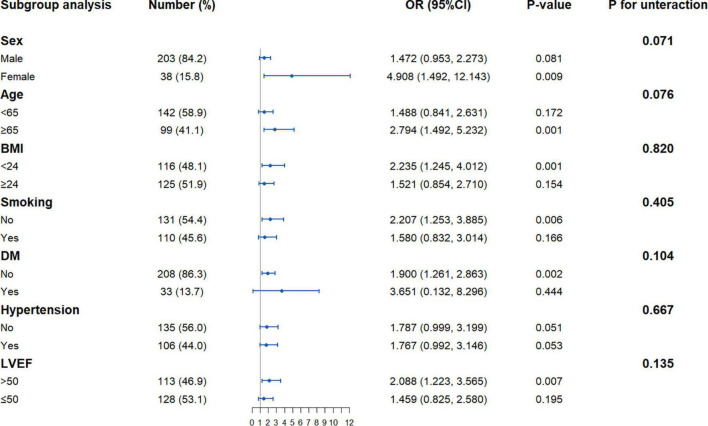
Forest plot investigating the association between the TyG index and with the risk of post-PCI QFR ≤ 0.92 in different subgroups. TyG, triglyceride-glucose; QFR, quantitative flow ratio; BMI, body mass index; LVEF, left ventricular ejection fraction; DM, diabetes mellitus; PCI, percutaneous coronary intervention; OR, odds ratio; CI, confidence interval.

The association of the TyG index with post-PCI QFR ≤ 0.92 was assessed in different subgroups (Figure 2). After adjusting for other factors, the positive association of the TyG index with the risk of post-PCI QFR ≤ 0.92 was more significant in the subgroups of women, age ≥ 65 years, BMI ≤ 24 kg/m^2^, non-smokers, no DM, and LVEF > 50%. In addition, there may be a slight interaction between the TyG index and male gender and age (Figure 2).

## Discussion

In the present study, we investigated the relationship between the TyG index and post-PCI QFR in STEMI patients undergoing PCI. The main findings of this study were as follows: (1) the TyG index was significantly correlated with post-PCI QFR ≤ 0.92 in STEMI patients. After adjusting for other confounding factors, the TyG index was still an independent risk factor for post-PCI QFR ≤ 0.92; (2) When the TyG index exceeds 9.75, the risk of post-PCI QFR ≤ 0.92 increases; (3) The positive association between the TyG index and the risk of post-PCI QFR ≤ 0.92 in STEMI patients was more significant in women, the older, non-smoking, no DM, and people with good cardiac function.

STEMI is a dangerous disease that seriously affects the life and quality of life of patients. With the development of interventional treatment technology, the prognosis of patients with STEMI has been improved, but one-quarter of patients still have adverse events during follow-up, which was related to residual coronary artery ischemia (1–3). Current coronary interventions aim to relieve anatomical stenosis, but coronary function after stenting has not been evaluated. A large number of recent studies have shown that QFR can well evaluate the physiological function of coronary arteries, and post-PCI QFR was significantly associated with future adverse cardiovascular events (7, 23). In the PANDA III trial, QFR ≤ 0.92 was the best cutoff value for predicting adverse cardiovascular events within 2 years, and after the QFR exceeded 0.92, increasing QFR had no effect on prognosis (8). Therefore, identifying the risk factors that affect the risk of QFR ≤ 0.92 after PCI has clinical value and can be used as an early intervention treatment method.

Insulin resistance refers to the lower-than-expected biological effect of insulin, manifested as a disturbance in the uptake and utilization of glucose. IR can not only induce chronic hyperglycemia, but also affect lipid metabolism and increase TG levels (24). The TyG index is a composite index composed of TG and FBG. It has been confirmed that the TyG index can be used as a substitute index for IR, which is a simpler and faster assessment of the body’s IR status (13, 25–27). Several studies have found that the TyG index is significantly associated with the occurrence of atherosclerotic cardiovascular disease (28–31). A cohort study found that, in long-term follow-up, the TyG index can identify people at high risk for cardiovascular events (28). At the same time, a meta-analysis summarizing multiple cohort studies found that after adjusting for the effects of age, gender, and DM, the TyG index was still independently associated with the risk of cardiovascular disease (29). In a RCSCD-TCM study, Su et al. conducted a retrospective analysis of 731 patients with CAD and found that the TyG index was associated with the severity of CAD, and an elevated TyG index could increase the risk of coronary multivessel disease (30). Alessandra et al. analyzed baseline data from patients in secondary cardiac care and found that the TyG index was associated with metabolic risk factors for the heart, and that a high level of the TyG index was more likely to develop symptomatic CAD (31). Moreover, the TyG index is considered to be a marker for identifying the risk of subclinical arteriosclerosis and is closely related to the degree of coronary artery calcification, carotid intima-media thickness, and brachial-ankle pulse wave velocity, which is not affected by traditional risk factors (32–36). In addition, recent evidence has also shown that the TyG index is not only an independent risk factor for stable CAD, but also has a positive correlation with poor prognosis in patients with acute myocardial infarction (18, 19). A study of 1,092 STEMI patients who underwent successful PCI for 1 year of follow-up found that high levels of TyG index increased the risk of cardiovascular adverse events during follow-up (18). Zhu et al. investigated the occurrence of in-stent stenosis in 1,574 patients with acute coronary syndrome during 1-year follow-up after stenting and found that the increase in the TyG index level was independently associated with in-stent stenosis (19). However, no study has investigated the relationship between TyG index and post-PCI QFR in STEMI patients. In the present study, we found that the TyG index was associated with residual coronary ischemia, as the higher the TyG index level, the greater the risk of post-PCI QFR ≤ 0.92. Therapeutic measures to lower the TyG index may be beneficial in reducing residual coronary ischemia in the future. Meanwhile, we also found that the risk of post-PCI QFR ≤ 0.92 was increased when the TyG index level exceeded 9.75, which may serve as a threshold for assessing residual coronary ischemia. In addition, the results of subgroup analysis showed that the TyG index and post-PCI QFR were also stable in different subgroups. Unexpectedly, this relationship was more significant in women, non-smoking patients, and non-DM patients. Although the exact mechanism is unclear, it is also a factor that we need to consider together.

The exact mechanism between the TyG index and post-PCI QFR in STEMI patients remains unclear, but this association may be based on IR status as assessed by the TyG index. First, IR can damage coronary endothelial function through oxidative stress and inducing inflammation (10). Second, DM is related to coronary vascular dysfunction, which may damage microcirculatory vasodilation and reduce coronary blood flow. IR is an important pathophysiological pathway leading to DM, and the two may have commonalities in the physiological and structural damage of coronary arteries (37–40). Third, IR in patients with acute myocardial infarction promotes local platelet activation and thrombin generation, increasing coronary thrombus burden, which may explain this relationship (41, 42). There may be more studies in the future to clarify the relationship between TyG index and post-PCI QFR.

This study has important clinical value to explore the relationship between the TyG index and the risk of post-PCI QFR ≤ 0.92. First, TyG index is a risk factor for post-PCI QFR ≤ 0.92, and controlling the level of TyG index may reduce residual ischemia in coronary arteries after PCI. Second, the TyG index is a conveniently measurable, easily accessible, and reproducible blood index that can assess patient coronary physiological function in real time during follow-up. Finally, TyG index above 9.75 increases the risk of post-PCI QFR ≤ 0.92, and 9.75 may be used as a threshold for the need for intensive drug therapy to improve coronary ischemia after PCI.

At the same time, there are some limitations in our study. First, this was a single-center study with limited sample size and limited generalization of the results. Second, the present study lacked a comparison of the HOMA-IR and the TyG index. Finally, the TyG index during hospitalization was only assessed once, and the dynamic changes of the TyG index were lacking.

## Conclusion

The TyG index was independently associated with the risk of post-PCI QFR ≤ 0.92 in STEMI patients. Meanwhile, when the TyG index exceeded 9.75, the risk of post-PCI QFR ≤ 0.92 increased.

## Data availability statement

The raw data supporting the conclusions of this article will be made available by the authors, without undue reservation.

## Ethics statement

The studies involving human participants were reviewed and approved by the Ethics Committee of Guangdong Provincial People’s Hospital. The patients/participants provided their written informed consent to participate in this study.

## Author contributions

BY: manuscript preparation and writing—original draft. XH, WW, JL, JJ, and ZL: data collection and collation. BY, YM, and WW: data analysis. CRLB and HD: writing—critical revisions. HD and YZ: conceptualization and approval of the final version of the manuscript for submission. All authors read and approved the final manuscript.
