# Supplementary material for: Triglyceride-glucose index is associated with quantitative flow ratio in patients with acute ST-elevation myocardial infarction after percutaneous coronary intervention
Source: Front Cardiovasc Med. 2022 Sep 8;9:1002030. doi: 10.3389/fcvm.2022.1002030 (PMC9493184; doi:10.3389/fcvm.2022.1002030)
Supplement: Supplementary file 1 [file Data_Sheet_1.docx]

Supplementary Table 1 Baseline characteristics.

|  | Total  (N=236) | Post-PCI QFR≤0.92  (n=88) | Post-PCI QFR>0.92  (n=148) | P value |
| --- | --- | --- | --- | --- |
| Age, years | 62±12 | 61±11 | 62±12 | 0.184 |
| Male | 198 (83.9) | 76 (86.2) | 122 (82.4) | 0.469 |
| BMI, kg/m^2^ | 24.21±3.55 | 24.32±3.80 | 24.15±3.40 | 0.712 |
| LVEF, % | 49.6±11.2 | 47.1±11.8 | 51.1±10.6 | **0.009^*^** |
| Medical history, n (%) |  |  |  |  |
| Smoking | 109 (46.2) | 37 (42.0) | 72 (48.6) | 0.517 |
| Hypertension | 103 (43.6) | 42 (47.7) | 61(41.2) | 0.345 |
| DM | 31 (13.1) | 11 (12.5) | 20 (13.5) | 1.000 |
| Family history of CAD | 4(1.7) | 1(1.1) | 3(2.0) | 1.000 |
| Previous myocardial infarction | 22 (9.3) | 8 (9.1) | 14(9.5) | 1.000 |
| Peripheral vascular disease | 5 (2.1) | 0 (0) | 5 (3.3) | 0.160 |
| O to D, minutes | 9.31±11.63 | 9.42±13.21 | 9.25±10.65 | 0.912 |
| STEMI types |  |  |  | **0.005*** |
| Anterior | 122 (51.7) | 56 (63.6) | 66 (44.6) |  |
| Non-anterior | 114 (48.3) | 32 (36.4) | 82 (55.4) |  |
| Laboratory tests |  |  |  |  |
| FBG, mmol/L | 8.76±4.01 | 9.62±4.53 | 8.25±3.59 | **0.017^*^** |
| Creatine, mmol/L | 87.02±73.65 | 81.11±34.30 | 90.54±89.12 | 0.342 |
| TG, mmol/L | 2.21±1.76 | 2.44±1.96 | 2.08±1.63 | 0.130 |
| TC, mmol/L | 4.58±1.73 | 4.52±1.91 | 4.61±1.62 | 0.702 |
| HDL-C, mmol/L | 1.08±0.28 | 1.04±0.22 | 1.10±0.30 | 0.135 |
| LDL-C, mmol/L | 3.37±0.93 | 3.43±1.07 | 3.32±0.83 | 0.371 |
| Peak CK-MB, U/L | 285.08±392.78 | 246.98±218.36 | 307.74±465.86 | 0.251 |
| Peak TnT, pg/mL | 8379.8±45990.0 | 5285.9±3432.3 | 10129.4±58009.8 | 0.427 |
| Peak NT-proBNP, pg/mL | 3232.0±8119.1 | 2964.0±4909.9 | 3391.3±9541.8 | 0.697 |
| TyG index | 9.32±0.79 | 9.50±0.83 | 9.21±0.75 | **0.005^*^** |

PCI, percutaneous coronary intervention; QFR, quantitative flow ratio; BMI, body mass index; LVEF, left ventricular ejection fraction; DM, diabetes mellitus; CAD, coronary artery disease; O to D, time from onset to door; STEMI, ST-elevation myocardial infarction; FBG, fasting blood glucose; TG, triglyceride; TC, total cholesterol; HDL-C, high-density lipoprotein cholesterol; LDL-C, low-density lipoprotein cholesterol; CK-MB, peak creatine kinase isoenzyme MB; TnT, troponin T; NT-proBNP , N terminal pro B type natriuretic peptide; TyG, triglyceride-glucose.

Supplementary Table 2 Coronary characteristics.

|  | Total  (N=236) | Post-PCI QFR≤0.92  (n=88) | Post-PCI QFR>0.92  (n=148) | P value |
| --- | --- | --- | --- | --- |
| Culprit vessel |  |  |  | **0.014*** |
| LAD | 121 (51.3) | 56 (63.6) | 65 (43.9) |  |
| LCX | 14 (5.9) | 4 (4.5) | 10 (6.8) |  |
| RCA | 101 (42.8) | 28 (31.8) | 73 (49.3) |  |
| Pre-PCI QCA |  |  |  |  |
| Reference vessel diameter, mm | 2.95±0.87 | 2.90±0.84 | 2.98±0.88 | 0.480 |
| Minimal lumen diameter, mm | 1.07±0.60 | 1.07±0.57 | 1.07±0.61 | 0.976 |
| Diameter stenosis, % | 63.2±17.0 | 62.9±16.0 | 63.5±17.6 | 0.799 |
| lesion length, mm | 14.3±6.5 | 13.5±5.4 | 14.9±7.0 | 0.086 |
| Post-PCI QCA |  |  |  |  |
| In-stent reference vessel diameter, mm | 3.12±0.62 | 3.04±0.52 | 3.17±0.67 | 0.106 |
| In-stent minimal lumen diameter, mm | 2.55±0.59 | 2.40±0.55 | 2.64±0.60 | **0.003*** |
| In-stent diameter stenosis, % | 18.5±11.3 | 21.0±13.5 | 17.0±9.5 | **0.017*** |
| Stent length, mm | 29.2±15.9 | 27.6±15.8 | 30.1±15.9 | 0.235 |
| Pre-PCI QFR | 0.40±0.39 | 0.38±0.37 | 0.41±0.41 | 0.619 |
| Post-PCI QFR | 0.93±0.08 | 0.87±0.11 | 0.96±0.02 | **<0.001*** |

LAD, left anterior descending artery; LCX, Left circumflex artery; RCA, right coronary artery; QCA, quantitative coronary angiography; PCI, percutaneous coronary intervention; QFR, quantitative flow ratio.

Supplementary Table 3 Association of TyG index with the risk of post-PCI QFR ≤ 0.92 in logistic regression models.

|  | OR | 95%CI | P value |
| --- | --- | --- | --- |
| Unadjusted model |  |  |  |
| TyG, per 1-unit increase | 1.620 | 1.145 to 2.292 | **0.006*** |
| Model 1 |  |  |  |
| TyG, per 1-unit increase | 1.620 | 1.145 to 2.292 | **0.006*** |
| Model 2 |  |  |  |
| TyG, per 1-unit increase | 1.672 | 1.171 to 2.369 | **0.005*** |
| Model 3 |  |  |  |
| TyG, per 1-unit increase | 1.687 | 1.162 to 2.448 | **0.006*** |

Model 1: adjusted for age, sex, and BMI

Model 2: adjusted for age, sex, BMI, LVEF, smoking, hypertension, DM, previous myocardial infarction, and creatine.

Model 3: adjusted for age, sex, BMI, LVEF, smoking, hypertension, DM, previous myocardial infarction, creatine, culprit vessel, length of stents, in-stent minimal lumen diameter and in-stent diameter stenosis.

TyG, triglyceride-glucose; OR, odds ratio; CI, confidence interval; BMI, body mass index; LVEF, left ventricular ejection fraction; DM, diabetes mellitus; PCI, percutaneous coronary intervention; QFR, quantitative flow ratio.

Supplementary Figure 1. Multivariable adjusted OR for the risk of post-PCI QFR ≤ 0.92 according to levels of TyG index on a continuous scale.


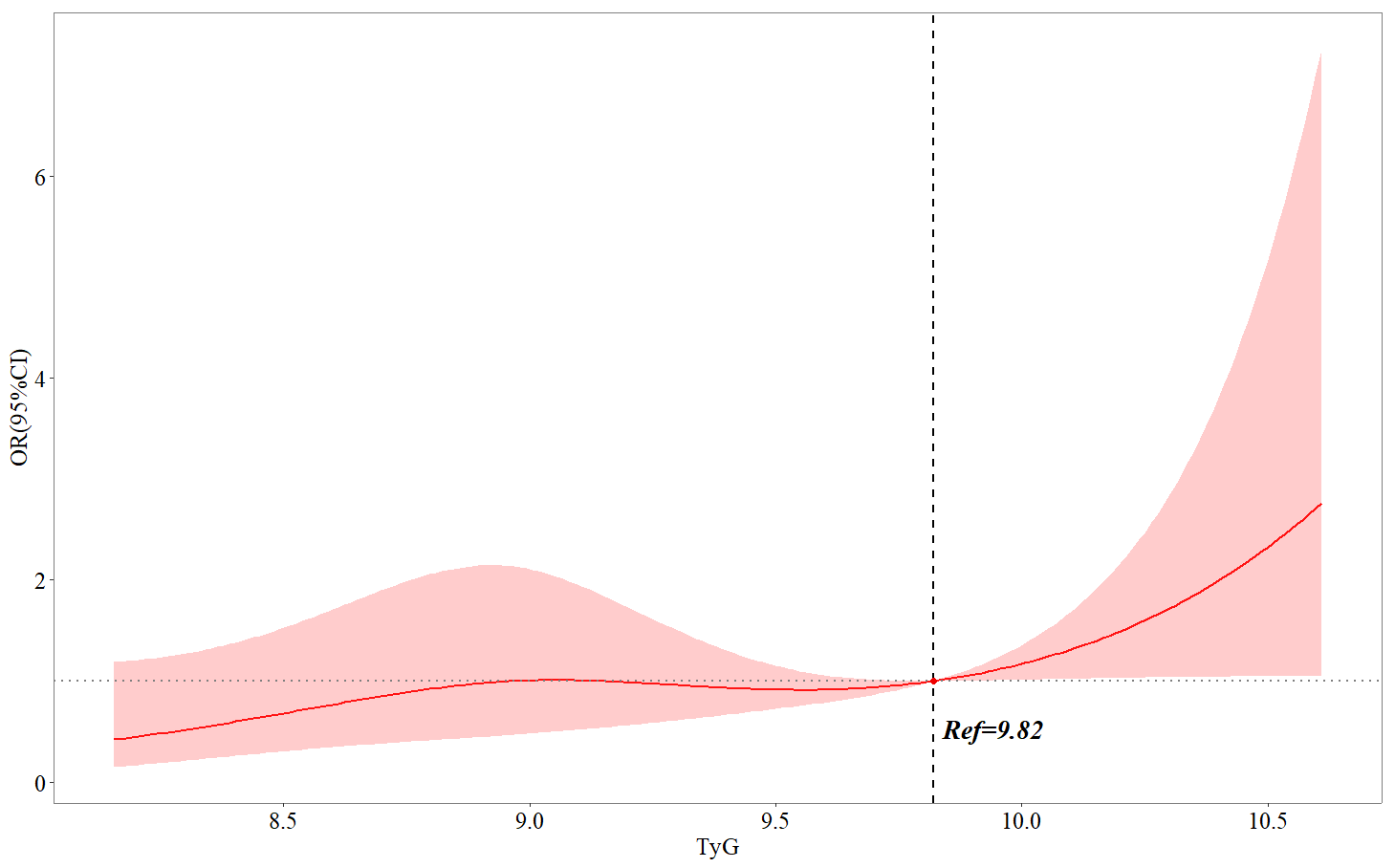


Odds ratios and 95% CIs derived from restricted cubic spline regression, with knots placed at the 5th, 35th, 65th, and 95th percentiles of the distribution of TyG index. The reference point for TyG index is located at OR=1. Ref represents the level of TyG index at increased risk of post-PCI QFR ≤ 0.92.

Analyses were adjusted for age, sex, BMI, LVEF, smoking, hypertension, DM, previous myocardial infarction, creatine, culprit vessel, length of stents, in-stent minimal lumen diameter and in-stent diameter stenosis.

OR: odds ratio; QFR, quantitative flow ratio; TyG: triglyceride-glucose; BMI, body mass index; LVEF, left ventricular ejection fraction; DM, diabetes mellitus; PCI, percutaneous coronary intervention; CI: confidence interval.

Supplementary Figure 2. Forest plot investigating the association between the TyG index and with the risk of post-PCI QFR ≤ 0.92 in different subgroups.


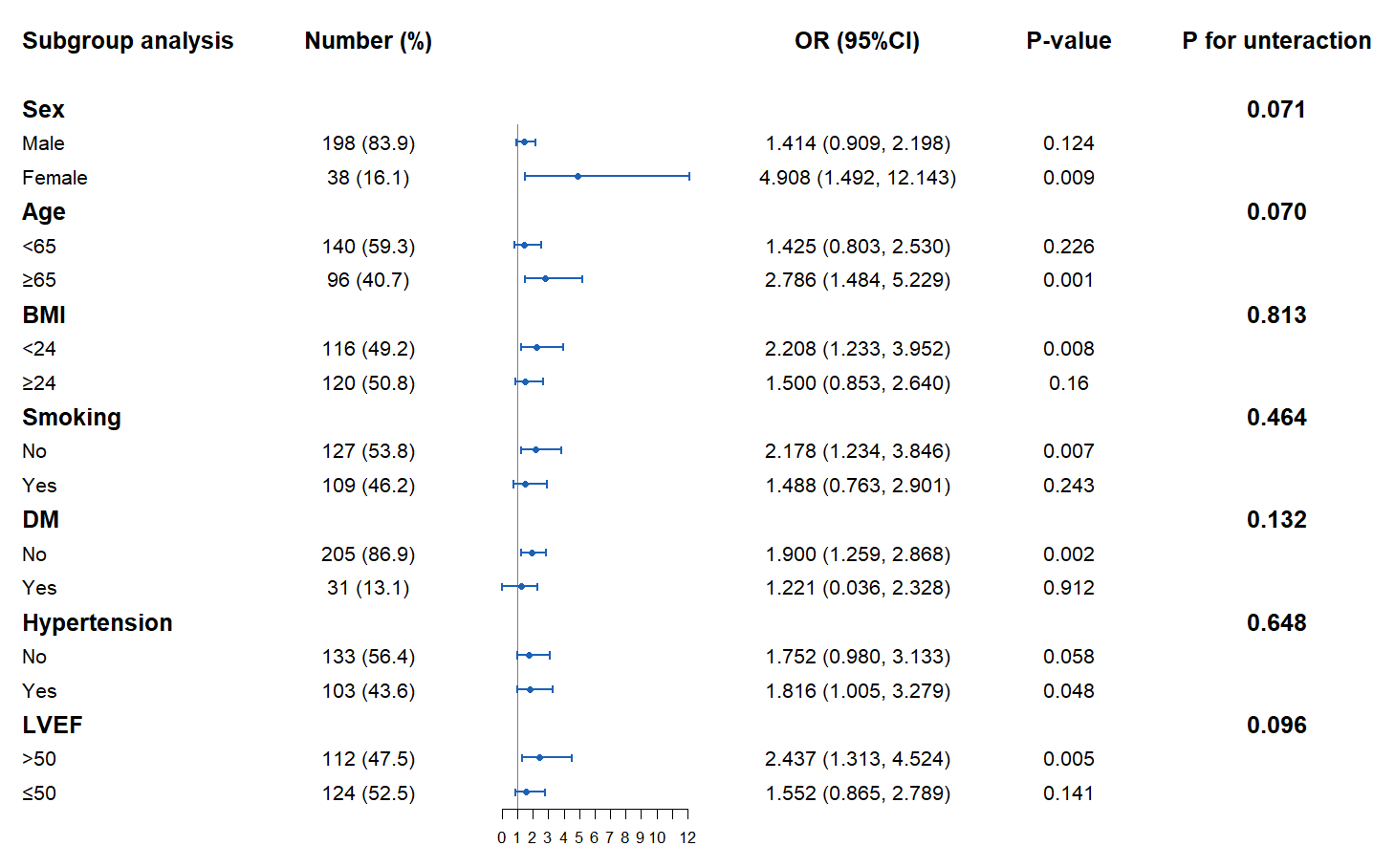


TyG: triglyceride-glucose; QFR, quantitative flow ratio; BMI, body mass index; LVEF, left ventricular ejection fraction; DM, diabetes mellitus; PCI, percutaneous coronary intervention; OR: odds ratio; CI: confidence interval.
